# Bioinformatics identification of potentially involved microRNAs in Tibetan with gastric cancer based on microRNA profiling

**DOI:** 10.1186/s12935-015-0266-1

**Published:** 2015-12-12

**Authors:** Yushuang Luo, Chengwu Zhang, Feng Tang, Junhui Zhao, Cunfang Shen, Cheng Wang, Pengjie Yu, Miaozhou Wang, Yan Li, J. I. Di, Rong Chen, Ge Rili

**Affiliations:** Research Center for High Altitude Medicine, Qinghai University of Medical School, Kunlong Road 16, Xining, 810001 China; Department of Oncology, Affiliated Hospital of Qinghai University, Xining, 810001 China; Department of Gastrointestinal Surgery, Affiliated Hospital of Qinghai University, Xining, 810001 China

**Keywords:** Gastric cancer, miRNA microarray, Differentially expressed miRNAs, Enrichment analysis, Network of miRNAs-targets

## Abstract

**Objective:**

The incidence of gastric cancer is high in Chinese Tibetan. This study aimed to identify the differentially expressed microRNAs (miRNAs) and further explore their potential roles in Tibetan with gastric cancer so as to predict potential therapeutic targets.

**Methods:**

A total of 10 Tibetan patients (male:female = 6:4) with gastric cancer were enrolled for isolation of matched gastric cancer and adjacent non-cancerous tissue samples. Affymetrix GeneChip microRNA 3.0 Array was employed for detection of miRNA expression in samples. Differential expression analysis between two sample groups was analyzed using Limma package. Then, MultiMiR package was used to predict targets for miRNAs. Following, the target genes were put into DAVID (Database for Annotation, Visualization and Integrated Discovery) to identify the significant pathways of miRNAs.

**Results:**

Using Limma package in R, a total of 27 differentially expressed miRNAs were screened out in gastric cancer, including 25 down-regulated (e.g. hsa-miR-148a-3p, hsa-miR-148b-3p and hsa-miR-363-3p) and 2 up-regulated miRNAs. According to multiMiR package, a number of 1445 target genes (e.g. *Wnt1*, *KLF4* and *S1PR1*) of 13 differentially expressed miRNAs were screened out. Among those miRNAs, hsa-miR-148a-3p, hsa-miR-148b-3p and hsa-miR-363-3p were identified with the most target genes. Furthermore, three miRNAs were significantly enriched in numerous common cancer-related pathways, including “Wnt signaling pathway”, “MAPK signaling pathway” and “Jak-STAT signaling pathway”.

**Conclusions:**

The present study identified a downregulation and enrichment in cancer-related pathways of hsa-miR-148a-3p, hsa-miR-148b-3p and hsa-miR-363-3p in Tibetan with gastric cancer, which can be suggested as therapeutic targets.

**Electronic supplementary material:**

The online version of this article (doi:10.1186/s12935-015-0266-1) contains supplementary material, which is available to authorized users.

## Background

Gastric cancer is one of the most common malignancies worldwide. Although incidence and mortality of gastric cancer has been dramatically decreased over the last decades worldwide, the declines are becoming less remarkable in some countries [1]. The main reason may be that there are limited treatment options and patients in advanced stages could not be cured by surgical removal [2]. Moreover, 5-year survival rates of gastric cancer are decreased with gastric cancer progression [3] and metastatic stage could lead to poor outcomes [4]. In Chinese Qinghai, the Tibetan ethnic group has a higher incidence of gastric cancer than the Han ethnic group [5]. Thus, there is an urgent need to develop effective treatments to improve diagnosis and reduce burden of gastric cancer in gastric cancer-infected Tibetan.

A number of key genes with abnormal expression in the progression of gastric cancer have been screened out. For example, the over-expression of *SPAG9* (sperm associated antigen 9) correlates with poor prognosis and leads to gastric cancer invasion and chemo-resistance [6]. In terms of Tibetan with gastric cancer, the expression pattern of tumor-associated antigen MG(7)-Ag is abnormal and it can be used as a reliable marker to predict gastric cancer at early stage [7]. The polymorphisms of prostate stem cell antigen gene are also associated with gastric cancer in Tibetans [8]. Therefore, the identification of key genes can improve diagnosis and management of gastric cancer in Tibetan.

MicroRNAs (miRNAs) are a group of small non-coding RNAs that have important roles in the development of numerous cancer types, through down-regulation of the target genes [9, 10]. Multiple miRNAs are expressed aberrantly and are involved in the progression and prognosis of gastric cancer [11]. Therefore, investigating role of miRNAs in gastric cancer could provide new insight into the biological mechanism of this disease. Reportedly, miR-21 is up-regulated in gastric cancer and its dysregulation can enhance cell proliferation, invasion and migration through down-regulating a set of tumor suppressor genes, such as *RECK* (reversion-inducing-cysteine-rich protein with kazal motifs) [12]. In addition, miR-544a could activate the Wnt signaling pathway by stabilizing the β-catenin in nucleus and its inhibition may be a therapeutic method for metastatic gastric cancer [13]. However, the research on miRNAs in gastric cancer in Tibetan is really rare and therefore, the exploration of miRNAs in the progression of gastric cancer in Tibetan is of great significance.

In the present study, the paired cancerous and adjacent non-cancerous tissue samples were collected from 10 patients with gastric cancer, and further conducted for miRNA expression profiling analysis. Differentially expressed miRNAs (DE-miRs) were screened out between two sample groups, followed by identification of target genes based on bioinformatics methods. Furthermore, functional enrichment analysis was performed for the DE-miRs so as to reveal their potential roles in progression of gastric cancer.

## Methods

### Sample collections

A total of 10 Tibetan patients (male:female = 6:4) with gastric cancer were enrolled in this study. They were aged between 33 and 77 years old, and the median age was 51.1. The tumor node metastasis stages (TNM) were determined basing on the International Union Against Cancer and the American Joint Committee on Cancer pathological staging systems. The patients were identified with clinical stages at T2N0M0(1/10), TisN0M0, TisN0M0IIc, TisN0M0IIc, T3N2M0, T3N0M0, T4aN1M0, T2N1M0, T3N2M0 or T3N2M0 (Table 1). Matched gastric cancer and adjacent non-cancerous tissue samples (n = 10 in each group) were obtained during surgical operation and immediately stored at −80 °C for microarray analysis. All the enrolled patients have given written informed consent and the present study was approved by Ethics Committee of Qinghai University Affiliated Hospital.

**Table 1:** Information on sample cases

Number	Age	Sex	Elevation (m)	Staging	HP infection	Pathological description
B1 299295	46	Male	2260	T2N0M0(1/10)		High-differentiated adenocarcinoma invaded submucosa, lymph node 0/10
B2 302629	38	Female	2850	TisN0M0		Intramucosal carcinoma in gastric antrum (0/11)
B3 WJ	57	Male	2840	T4aN1M0		Distal gastric carcinoma, tumor invaded full thickness and vascular nerves (+) 2/15, low and median differentiated adenocarcinoma
B5 211409	56	Male	2850	TisN0M0IIc		
B6	49	Male	2050	TisN0M0IIc		Intramucosal carcinoma
A1 303135	43	Male	3280	T3N2M0		Low differentiated tubular adenocarcinoma invaded deep muscle layer (6/9), vascular and nerve (+)
A2 302628	33	Male	3860	T3N0M0		Ulcer tubular well-differentiated adenocarcinoma invaded the deep muscularis serosa, vascular nerve (+), 0/13
A3 WJ	56	Female	3800	T2N1M0	HP−, gastroscope	Distal gastric cancer
A4 WJ	77	Female	4800	T3N2M0	HP+, gastroscope	Gastric carcinoma
A6 300804	56	Male	3100	T3N2M0		Differentiated tubular adenocarcinoma invaded deep muscularis, vascular+, nerve+

*HP* *Helicobacter pylori*

### Microarray profiling of miRNAs

Total RNA was extracted from the matched cancerous and adjacent non-cancerous tissues according to the manufacture’ s instructions using RNAiso Plus purchased from Treasure Biological Engineering (Dalian, China). Reverse transcription-quantitative polymerase chain reaction was conducted according to the manufacture’ s instructions using a PrimeScript^®^ First Strand cDNA Synthesis kit and miRNA qPCR primer mix (Takara Bio, Inc, CA, USA). Affymetrix GeneChip microRNA 3.0 Array (Affymetrix, Inc, Santa, CA, USA) was employed for detection of miRNA expression in samples, which provides for 100 % miRBase v17 coverage (http://www.mirbase.org) by a one-color approach.

### Differential expression analysis

Raw data of miRNA expression profile from cancerous and adjacent non-cancerous tissues were converted into recognizable miRNA expression data by RMA (robust multi-array analysis) method, followed by median normalization and log2 transformation using Affy package (http://www.bioconductor.org/packages/release/bioc/html/affy.html) in R [14]. During the expression conversion from probe level to miRNA level, the expression values of probes corresponding to each miRNA were averaged as the miRNA value. Differential expression analysis between two groups was analyzed using Limma package of R language (http://www.bioconductor.org/packages/release/bioc/html/limma.html) [15] based on the criteria of |log2 FC (fold change)| ≥1 and *P* value <0.05.

### Prediction of targets for differentially expressed miRNAs

MultiMiR package (http://multimir.ucdenver.edu/) [16] was previously established to predict targets of miRNAs, which covered 14 databases including miRecords, miRTarBase, TarBase, DIANA-microT, ElMMo, MicroCosm, miRanda, miRDB, PicTar, PITA, TargetScan, miR2Disease, PharmacomiR and PhenomiR. In the present study, multiMiR package was employed to predict targets of DE-miRs with the criterion of primary score listed in top 35. Meanwhile, only the target genes predicted in at least three databases were selected for following analysis. Accordingly, the network between targets and DE-miRs was constructed using Cytoscape v3.2.1 (http://www.cytoscape.org/) [17] software.

### Functional enrichment analysis for DE-miRs

Database for Annotation, Visualization and Integrated Discovery (DAVID, https://david.ncifcrf.gov/home.jsp) [18] is a powerful tool to mine functions of interested genes. As miRNAs function through down-regulating target genes, to further reveal the potential biological functions or pathways that may be changed by the DE-miRs, the target genes were put into DAVID to screen out significantly enriched Kyoto Encyclopedia of Genes and Genomes (KEGG, http://www.kegg.jp/) [19] pathways and Gene Ontology (GO, http://www.geneontology.org) [20] biological processes. The selection criterion for significant GO and KEGG pathway terms was *P* value <0.05.

## Results

### DE-miRs between gastric cancer and adjacent non-cancerous samples

**Fig. 1:**
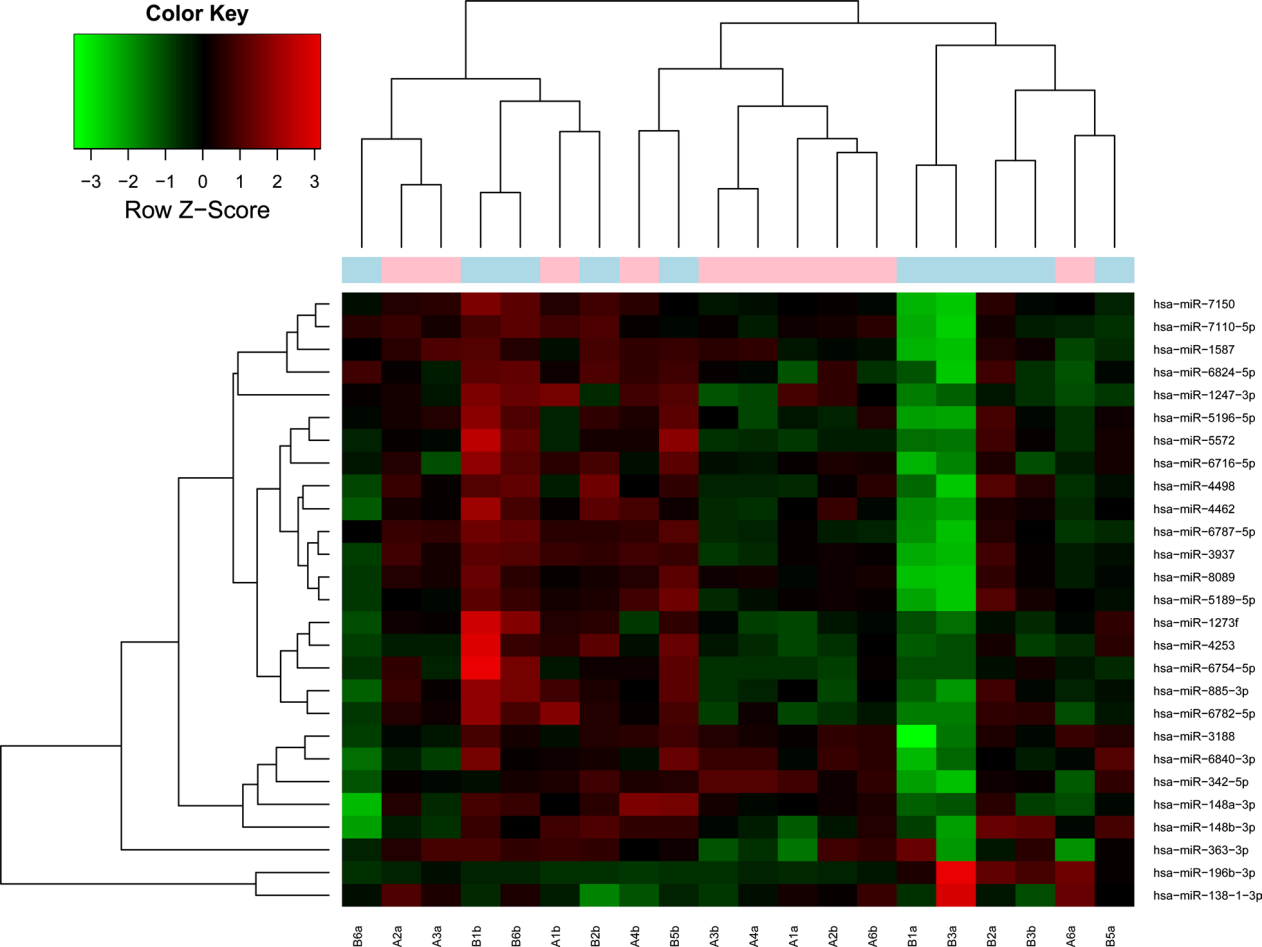
Heat map of differentially expressed microRNAs. *Red* represents up-regulation and *green* represents down-regulation

By using Limma package in R with the criteria of |log2 FC| ≥1 and *P* value <0.05, a total of 27 DE-miRs in gastric cancer were screened out, including 25 down-regulated miRNAs (e.g. hsa-miR-148a-3p, hsa-miR-148b-3p and hsa-miR-363-3p) and 2 up-regulated miRNAs (hsa-miR-196b-3p and hsa-miR-138-1-3p) (Table 2). The heap map of DE-miRs was shown in Fig. 1.

**Table 2:** The 27 differentially expressed microRNAs in gastric cancer

Accession	MicroRNAs	Log fold change	*P* value
MIMAT0000243	hsa-miR-148a-3p	−1.8142778	0.006134223
MIMAT0027583	hsa-miR-6840-3p	−1.47970911	0.015759083
MIMAT0027464	hsa-miR-6782-5p	−1.45651738	0.018393456
MIMAT0000759	hsa-miR-148b-3p	−1.43506791	0.010053602
MIMAT0028117	hsa-miR-7110-5p	−1.43359191	0.038837699
MIMAT0000707	hsa-miR-363-3p	−1.3662862	0.049770472
MIMAT0018352	hsa-miR-3937	−1.32835296	0.023596283
MIMAT0004694	hsa-miR-342-5p	−1.27792593	0.044159324
MIMAT0022721	hsa-miR-1247-3p	−1.17878373	0.020578891
MIMAT0019077	hsa-miR-1587	−1.1697371	0.048251207
MIMAT0016882	hsa-miR-4253	−1.15418525	0.017333874
MIMAT0027474	hsa-miR-6787-5p	−1.14471871	0.040401132
MIMAT0015070	hsa-miR-3188	−1.10659872	0.042107841
MIMAT0025844	hsa-miR-6716-5p	−1.08787366	0.019653906
MIMAT0019033	hsa-miR-4498	−1.08655973	0.018678343
MIMAT0004948	hsa-miR-885-3p	−1.0692305	0.044870796
MIMAT0018986	hsa-miR-4462	−1.0690462	0.004846197
MIMAT0028211	hsa-miR-7150	−1.06220727	0.040895785
MIMAT0027408	hsa-miR-6754-5p	−1.03580421	0.022410575
MIMAT0021128	hsa-miR-5196-5p	−1.0324015	0.035802131
MIMAT0020601	hsa-miR-1273f	−1.02885192	0.031420972
MIMAT0022260	hsa-miR-5572	−1.02726895	0.033720698
MIMAT0021120	hsa-miR-5189-5p	−1.01814356	0.020114063
MIMAT0027548	hsa-miR-6824-5p	−1.01710815	0.020467938
MIMAT0031016	hsa-miR-8089	−1.01329602	0.021980497
MIMAT0009201	hsa-miR-196b-3p	1.33573282	0.048038696
MIMAT0004607	hsa-miR-138-1-3p	1.405280355	0.023694469

Log fold change >0, up-regulated; log fold change <0, down-regulated

### Analysis of network of miRNAs-targets

**Fig. 2:**
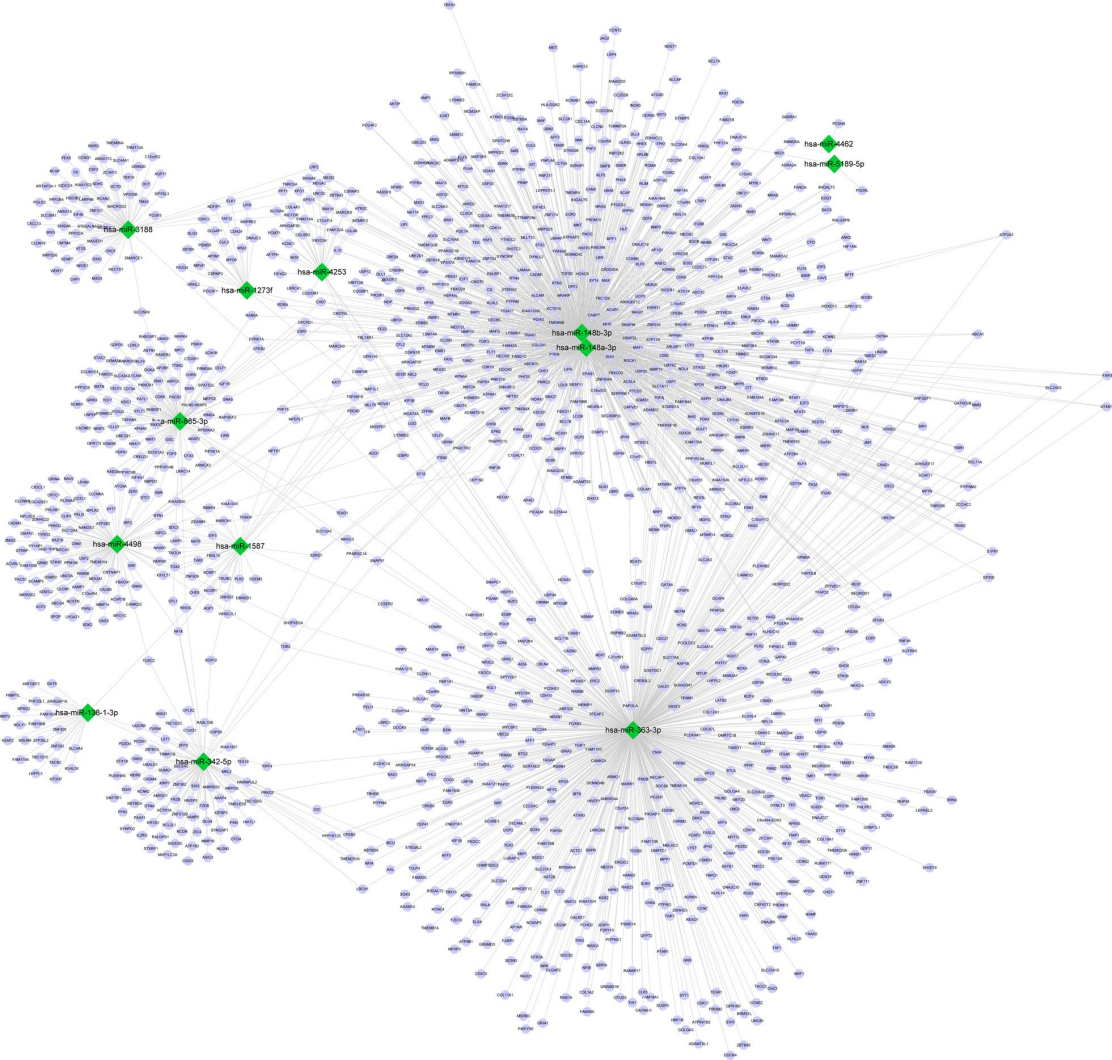
Network of differentially expressed microRNAs-target genes. *Circle* represents target genes and *diamond* represents microRNAs. *Red* represents up-regulation and *green* represents down-regulation

According to multiMiR package, a number of 1445 target genes of 13 DE-miRs were screened out. Based on the aforementioned criterion, the network of miRNA-target was constructed using Cytoscape (Fig. 2). In the network, hsa-miR-148a-3p, hsa-miR-148b-3p and hsa-miR-363-3p were identified with the most target genes, and furthermore, the three miRNAs targeted numerous common genes such as *CAND1* (cullin-associated and neddylation-dissociated 1), *KLF4* (Kruppel-like factor 4), *S1PR1* (sphingosine-1-phosphate receptor 1), *Wnt1* (wingless-type MMTV integration site family, member 1), *CNTN4* (contactin 4) and *BCL2L11* [BCL2-like 11 (apoptosis facilitator)].

### Functional and pathways of DE-miRs

**Fig. 3:**
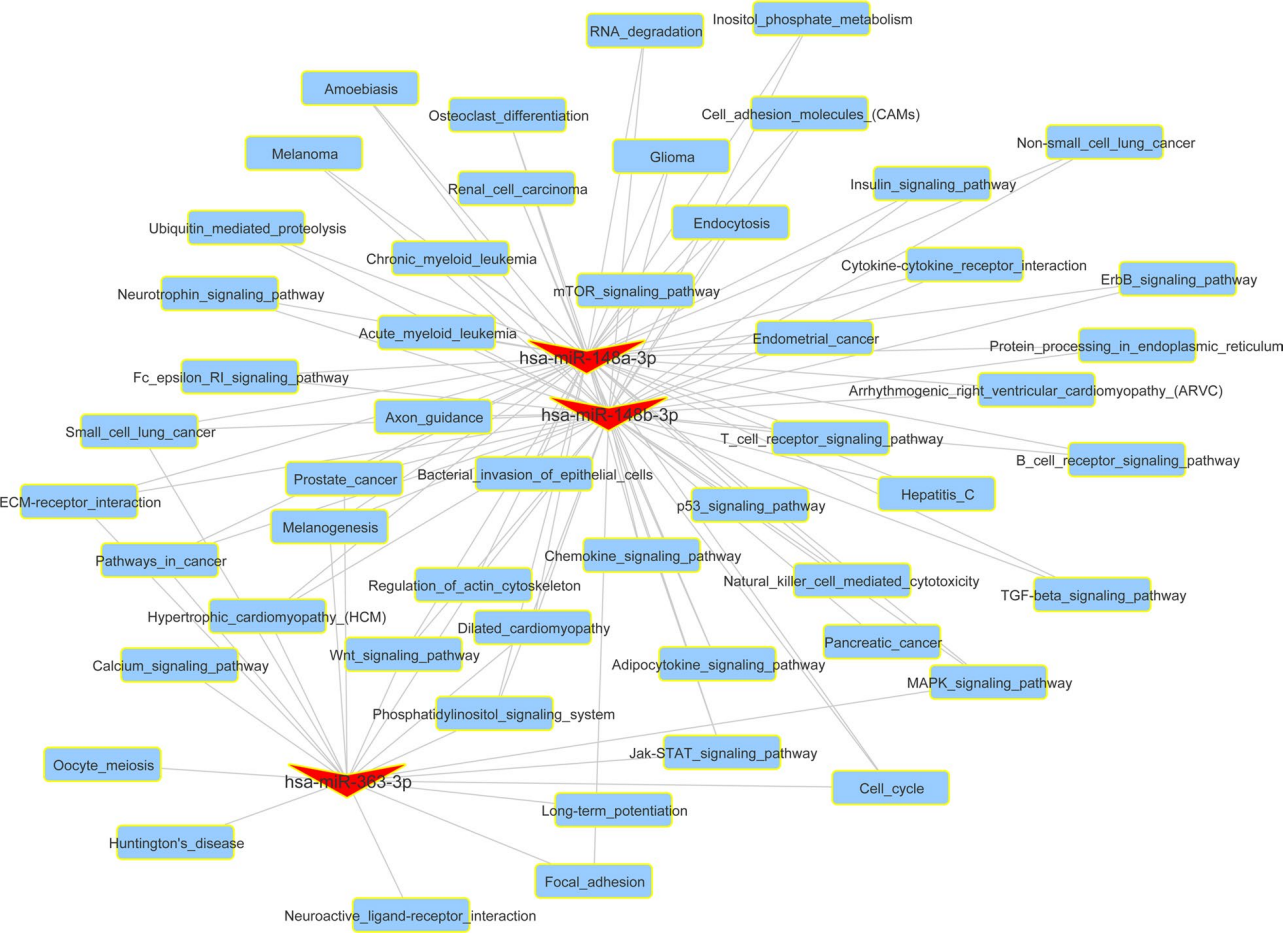
Network of functional enrichment results of differentially expressed microRNAs. *V-shape* represents microRNA and *rectangle* represents significant pathways enriched by target genes of microRNAs

KEGG pathway and GO functional enrichment analysis indicated that only three miRNAs (hsa-miR-148a-3p, hsa-miR-148b-3p and hsa-miR-363-3p) were significantly enriched in numerous common KEGG pathways and GO biological processes (*P* < 0.05, Additional file 1: Table S1). Among those significant pathways, some known cancer-related pathways were screened out, including “pathway in cancer”, “Wnt signaling pathway”, “MAPK signaling pathway” and “Jak-STAT signaling pathway” (Fig. 3).

## Discussion

MiRNAs exert regulatory effects on gene expression in humans, resulting in cell growth, differentiation and apoptosis via down-regulating target genes in cancer [21]. In the present study, a total of 27 DE-miRs were screened out, including hsa-miR-148a-3p, hsa-miR-148b-3p and hsa-miR-363-3p, which targeted the most genes. Furthermore, the three miRNAs were significantly enriched in cancer-related pathways, including Wnt signaling pathway, MAPK (mitogen-activated protein kinase) signaling pathway and Jak-STAT (Janus kinase-signal transducer and activator of transcription) signaling pathway.

Wnt signaling pathway is implicated in cancer development and its hyperactivation can lead to enhanced tumorigenicity and increased metastatic potential [22]. In gastric cancer, overexpressed miR-544a is demonstrated to activate WNT signaling pathway which further contributes to the disease progression [13]. In the present study, we identified that hsa-miR-148a-3p, hsa-miR-148b-3p and hsa-miR-363-3p were remarkably enriched in Wnt signaling pathway. Reportedly, miR-148a inhibits the metastasis of hepatocellular carcinoma via acting on Wnt signaling pathway [23]. MiR-363 down-regulates the expression of myeloid cell leukemia-1 [24] whose expression correlates with phosphorylated glycogen synthase kinase-3beta, the key component of Wnt signaling pathway in breast cancer [25]. Notably, the above miRNA targets, such as *Wnt1*, were significantly enriched in Wnt signaling pathway. We can speculate that the miR-148a, miR-148b and miR-363 may play significant roles in gastric cancer progression via regulating the Wnt signaling pathway.

Besides, MAPK signaling pathway is dysregulated in gastric cancer, leading to abnormal cell proliferation and metastasis [26]. Moreover, the signaling pathway is implicated in drug resistance in gastric cancer by regulating the expression of apoptotic proteins *Bax* (BCL2-associated X protein)/*Bcl*-*2* (B-cell CLL/lymphoma 2) [27]. Our enrichment analysis showed that hsa-miR-148a-3p, hsa-miR-148b-3p and hsa-miR-363-3p were also significantly enriched in MAPK signaling pathway. In breast cancer, miR-148a acts as a tumor suppressor via targeting *IGF*-*IR* (insulin-like growth factor-I receptor) and *IRS1* (insulin receptor substrate 1) and further suppressing the downstream MAPK signaling pathway [28]. Besides, miR-363 is found to be down-regulated in gastric cancer and its down-regulation is associated with tumor differentiation, TNM stage and lymph-node metastasis [29]. Notably, the suppression of their common target *KLF4* could inhibit the expression of various *Erk5* (mitogen-activated protein kinase 7) targets and further affect the MAPK cascade in the regulation of endothelial integrity in cancer [30]. The enrichment of miR-148a, miR-148b and miR-363 in MAPK signaling pathway may suggest a joint contribution to gastric cancer development via involving MAPK signaling pathway.

Additionally, hsa-miR-148a-3p, hsa-miR-148b-3p and hsa-miR-363-3p were found to be dramatically enriched in Jak-STAT signaling pathway. Jak-STAT serves as a straightforward mechanism whereby cells sense environmental cures and further regulate cell growth and differentiation in cancer [31]. The inhibition of Jak-STAT signaling pathway can lead to decreased cell proliferation and enhanced cell apoptosis in gastric cancer cells [32]. Exogenous miR-363 promotes cell growth, progression and tumorsphere formation of SC-M1 gastric cancer cells, and the knockdown of miR-363 suppresses tumorigenesis of SC-M1 cells [33]. MiR-148a/b is dysregulated and their haplotype is significantly correlated with gastric cancer [34]. *S1PR1*, one of the common targets of miR-148a-3p, -148b-3p and -363-3p, is implicated in *NFκB/IL*-*6/STAT3/S1PR1* amplification loop that is important for chronic colitis-related cancer and can be suggested as therapeutic option [35]. The enrichment of the three miRNAs in Jak-STAT signaling pathway implies their involvement in gastric cancer progression via Jak-STAT signaling pathway.

We should note that there were some limitations in the present study. Herein, although we demonstrated a significant enrichment of dysregulated hsa-miR-148a-3p, hsa-miR-148b-3p and hsa-miR-363-3p in cancer-related pathways in patients with gastric cancer, we did not further validate their expressions, nor demonstrate their roles in cancer-related pathways using systematically functional experiments. Moreover, as there are only 10 samples enrolled in this study, we did not further consider the DE-miRs among different stages. Besides, the present results were just obtained based on microarray analysis and bioinformatics prediction, and needed to be further validated in future. However, this study can be regarded as a preliminary study and to an extent provide some valuable directions for future studies, especially for researches on gastric cancer in Tibetan.

In summary, the present study identified a downregulation of hsa-miR-148a-3p, hsa-miR-148b-3p and hsa-miR-363-3p in gastric cancer in Tibetan using microarray analysis. What is more, we demonstrated their significant enrichment in cancer-related pathways, including Wnt signaling pathway, MAPK signaling pathway and Jak-STAT signaling pathway. These findings suggested the potential usage of hsa-miR-148a-3p, hsa-miR-148b-3p and hsa-miR-363-3p as diagnostic and therapeutic biomarkers for gastric cancer-infected Tibetan. However, further experimental validations are in urgent need to confirm these results.

## Additional file

10.1186/s12935-015-0266-1 The significant KEGG and GO_BP terms of differentially expressed microRNAs. KEGG, Kyoto Encyclopedia of Genes and Genomes; GO Gene Ontology; BP, biological processes.

## Abbreviations

*Bax*: BCL2-associated X protein
*Bcl*-*2*: B-cell CLL/lymphoma 2
*CAND1*: cullin-associated and neddylation-dissociated 1
*KLF4*: Kruppel-like factor 4
*S1PR1*: sphingosine-1-phosphate receptor 1
*Wnt1*: wingless-type MMTV integration site family, member 1
*CNTN4*: contactin 4
*BCL2L11*: BCL2-like 11 (apoptosis facilitator)
GO: Gene Ontology
MicroRNAs: miRNAs
*SPAG9*: sperm associated antigen 9
*RECK*: reversion-inducing-cysteine-rich protein with kazal motifs
TNM: tumor node metastasis stages
